# Therapeutic strategy using radiofrequency ablation for para‐Hisian accessory pathway

**DOI:** 10.1002/clc.24263

**Published:** 2024-03-27

**Authors:** Naoya Kataoka, Teruhiko Imamura

**Affiliations:** ^1^ Second Department of Internal Medicine University of Toyama Toyama Japan

To editor:

Chai and colleagues have demonstrated the safety and efficacy of catheter ablation for para‐Hisian accessory pathways, despite the inherent risk of conduction disorders due to their proximity to the cardiac conduction system.^1^ Notably, the superior vena cava (SVC) approach proved effective, particularly in cases where conventional ablation via the inferior vena cava (IVC) had previously failed. However, various concerns have arisen in this context, particularly with the absence of comparisons to previously proposed approaches, such as trans‐noncoronary cusp or cryoballoon ablation.

A primary risk associated with radiofrequency ablation of para‐Hisian accessory pathways is the potential for procedure‐induced atrioventricular block. Recent literature has favored cryoballoon ablation over conventional radiofrequency approaches due to its reduced invasiveness to the myocardium, although the therapeutic efficacy of cryoballoon ablation remains under debate.^2^ Notably, atrioventricular block necessitates pacemaker implantation and escalates the risk of severe complications, such as device‐related infections. Most arrhythmias stemming from para‐Hisian accessory pathways, such as atrioventricular reentrant tachycardia, do not bear a significant association with mortality. Therefore, a comprehensive evaluation of risks and benefits is imperative in choosing the appropriate energy source for ablation procedures.

Recent studies have highlighted the efficacy of using irrigated catheters for refractory accessory pathways that do not respond to conventional nonirrigated catheters.^3^ Irrigated catheters facilitate deeper tissue ablation, suggesting that radiofrequency ablation via a non‐coronary cusp approach—rather than the previously utilized SVC approach—could potentially address refractory para‐Hisian accessory pathways. Furthermore, the introduction of contact force and three‐dimensional mapping technologies has enhanced the precision of ablation. Employing this technology could potentially render radiofrequency ablation via the IVC approach effective in treating these pathways.

Numerous participants in the authors' study experienced failed ablation attempts via the IVC approach.^1^ The authors should delve further into the reasons why the SVC approach might be more effective compared to the IVC approach. The success rate of the procedure heavily relies on proper sheath selection. It is plausible that the authors utilized the type SL‐0 long sheath, which might not be optimal for ablating para‐Hisian accessory pathways, demanding a higher level of device system stability. Instead, the use of steerable sheaths could be preferable to ensure the requisite stability of the device system during the procedure. Additionally, specific details regarding the devices employed for radiofrequency ablation via the IVC approach for para‐Hisian accessory pathways remain undisclosed and warrant elucidation.
